# CXCL12/CXCR4 Axis Triggers the Activation of EGF Receptor and ERK Signaling Pathway in CsA-Induced Proliferation of Human Trophoblast Cells

**DOI:** 10.1371/journal.pone.0038375

**Published:** 2012-07-27

**Authors:** Hong-Bo Zhao, Chuan-Ling Tang, Yan-Li Hou, Li-Rong Xue, Ming-Qing Li, Mei-Rong Du, Da-Jin Li

**Affiliations:** 1 Laboratory for Reproductive Immunology, Hospital and Institute of Obstetrics and Gynecology, Fudan University Shanghai Medical College, Shanghai, China; 2 Department of Obstetrics and Gynecology, the Affiliated Hospital, Hainan Medical College, Haikou, China; Institute of Zoology, Chinese Academy of Sciences, China

## Abstract

**Introduction:**

Our previous study has demonstrated Cyclosporin A (CsA) promotes the proliferation of human trophoblast cells. Therefore, we further investigate the intracellular signaling pathway involved in the CsA-induced proliferation of human trophoblast cells.

**Methods:**

Enzyme-linked immunosorbent assay (ELISA) was performed to evaluate the regulation of CsA on CXCL12 secretion in human trophoblast cells. Immunofluorescence analysis and western blotting analysis were used to investigate the role of CXCL12/CXCR4 axis in the CsA-induced epidermal growth factor receptor (EGFR) phosphorylation in human trophoblast cells. 5-bromo-2′-deoxyuridine (BrdU) cell proliferation assay was performed to analyze the involvement of EGFR and its downstream extracellular signal-regulated protein kinase (ERK) signaling pathway in the CsA-induced proliferation of human trophoblast cells.

**Results:**

Low concentration of CsA promoted the secretion of CXCL12, and recombinant human CXCL12 promoted the phosphorylation of EGFR in primary human trophoblast cells and choriocarcinoma cell line JEG-3. The inhibition of CXCL12 or CXCR4 by either neutralizing antibodies or small interfering RNA (siRNA) could completely block the CsA-induced EGFR phosphorylation. The CsA-induced proliferation of human trophoblast cells was effectively abrogated by the EGFR inhibitor AG1478 as well as the ERK inhibitor U0126, but not by the PI3K/PKB inhibitor LY294002. CsA promoted the activation of ERK in JEG-3 cells, which was markedly abrogated in the presence of CXCL12 siRNA, or CXCR4 siRNA, or AG1478.

**Conclusions:**

CsA may promote EGFR activation via CXCL12/CXCR4 axis, and EGFR downstream ERK signaling pathway may be involved in the CsA-induced proliferation of human trophoblast cells.

## Introduction

Cyclosporin A (CsA) is a potent immunosuppressive agent that is classically used in organ transplantation to prevent from allograft rejection. It exerts immunosuppressive effects mainly by binding to its cytoplasmic receptor cyclophilin A, inhibiting calcium-dependent calcineurin (CaN) activation, thus blocking the activation of nuclear factor of activated T cells (NFAT), and resulting in the inhibition of lymphokine genes essential for T-cell proliferation and activation [1]–[3].

Our previous study *in vivo* has demonstrated that administration with CsA at early stage of pregnancy successfully decreases the embryo resorption rate in the abortion-prone CBA/J×DBA/2 matings [4]. The study *in vitro* has provided evidence that CsA at low concentration promotes the proliferation and invasion of human first-trimester trophoblast cells through mitogen-activated protein kinase 3 (MAPK3)/MAPK1 [5]–[7]. Further study has demonstrated that CsA at low concentration down-regulates E-cadherin expression through EGFR/ERK signaling pathway, and ultimately improves the invasion of human trophoblast cells [8]. Epidermal growth factor (EGF) and its receptor (EGFR) are frequently highly activated in placenta, and play pivotal roles in the regulation of proliferation in human trophoblast cells [9]. It remains elusive how EGFR signaling pathway is activated by CsA and whether EGFR cascade is involved in the CsA-induced proliferation of human trophoblast cells.

CXC motif chemokine 12 (CXCL12), also known as stromal cell-derived factor-1 (SDF-1), is a small cytokine belonging to the chemokine family that exerts its effects by binding to its receptor CXCR4, a member of the G-protein-coupled receptor superfamily [10], [11]. Their interaction has been reported to be unique, different from other chemokines that recognize multiple receptors [12], [13]. CXCL12 was initially cloned and identified as pre-B-cell growth-stimulating factor [14], but its functions have been found to far beyond B cell lymphopoiesis, including T cell activation and migration, organ vascularization, hematopoiesis, neuronal development, immune cell homing and trafficking, and tumorigenesis [15]–[18]. Our previous study has shown that CXCL12 is expressed in human trophoblast cells, and promotes the proliferation of human trophoblast cells in a dose-dependent manner [19].

In the present study, we showed that CsA promoted the secretion of CXCL12, and recombinant human CXCL12 promoted the phosphorylation of EGFR in human trophoblast cells. The CsA-induced EGFR activation could be blocked by the neutralizing antibody or siRNA against CXCL12 or CXCR4. Further data showed that CsA improved the proliferation of human trophoblast cells through EGFR downstream ERK, rather than PI3K/PKB. These data described a detailed signaling pathway responsible for the CsA-induced proliferation of human trophoblast cells.

## Methods

### Reagents and antibodies

Monoclonal antibodies to EGFR, phosphorylated-EGFR (Tyr 1173), CXCR4, neutralizing antibodies against CXCL12 and CXCR4, FITC conjugated secondary antibody, and glyceraldehyde-3-phosphate dehydrogenase (GAPDH) were purchased from Santa Cruz. Recombinant human CXCL12, U0126, LY294002, and AG1478 were obtained from Sigma-Aldrich. PE-conjugated secondary antibody was purchased from R&D systems (Minneapolis, MN). Secondary antibodies conjugated with HRP were purchased from Kang-Chen Biotech (Shanghai, China).

Human CXCL12 immunoassay kit was purchased from R&D systems (Minneapolis, MN). The BrdU cell proliferation assay kit was purchased from Millipore (MA, USA). CXCL12 siRNA, CXCR4 siRNA, non-targeting siRNA control, and Lipofectamine 2000 reagent were obtained from Invitrogen (Carlsband, CA).

### Isolation and primary culture of human first-trimester trophoblast cells

The first-trimester human placental tissue (6–9 wk of gestation) was obtained from normal pregnancy, which was terminated for nonmedical reasons. The study has been approved by the Human Research Ethics Committee of Obstetrics and Gynecology Hospital, Fudan University, and each patient completed a signed, written consent form. The trophoblast cells were isolated by the trypsin-DNase I digestion and discontinuous Percoll gradient centrifugation, as described by our previous study [20]. The isolated human trophoblast cells were cultured in Dulbecco modified Eagle medium (DMEM)-high glucose complete medium (2 mM glutamine, 25 mM HEPES, 100 IU/ml penicillin, and 100 μg/ml streptomycin) supplemented with 15% fetal bovine serum (FBS, Gibco, Grand Island, NY), and incubated in 5% CO_2_ at 37°C. Cytokeratin 7 (CK-7) is currently regarded as the marker for trophoblast cells [21]. The isolated trophoblast cells were stained positively for CK-7 but negatively for vimentin. The purity of the isolated trophoblast cells was >95%. The human choriocarcinoma cell line JEG-3 was obtained from the cell bank at the Chinese Academy of Sciences (Shanghai, China) with the original source being the American Type Culture Collection (ATCC) (Manassas, VA, USA) and cultured in 1640 complete medium supplemented with 10% FBS in 5% CO_2_ at 37°C.

### RNA isolation and RT-PCR

Total RNAs were isolated by using the Trizol system (Watson Biotechnologies, Shanghai, China) according to the manufacturer's guidelines. Oligo dT primer and MMLV-RTase were used for the first strand synthesis. The cDNA products (2 μl) were mixed with Taq DNA polymerase (SABC, Luoyang, China), 50 pmol/l of each appropriate primer, 200 μmol/l each dNTP in a reaction buffer containing 10 mmol/l Tris-HCl (pH 8.3), 50 mmol/l KCl, 0.01% (W/V) bovine serum albumin (BSA), 2 mmol/l MgCl_2_ in a final volume of 100 μl. The primers used for the detection of CXCL12, CXCR4, and GAPDH were indicated in Table 1. The samples were amplified for 30 cycles at cyclic temperatures of 94°C 30s, 55°C 30s, 72°C 1 min. PCR products were analyzed through 2% agarose gel electrophoresis and ethidium bromide staining.

**Table 1:** Primer sequences used for the RT-PCR.

Gene	Sequences	Product size (bp)
CXCL12	F: 5′-ATGAACGCCAAGGTCGTGGTCG-3′	202
	R: 5′-TGTTGTTGTTCTTCAGCCG-3′	
CXCR4	F: 5′-GAACTTCCTATGCAAGGCAGTCC-3′	302
	R: 5′-CCATGATGTGCTGAAACTGGAAC-3′	
GAPDH	F: 5′-GGGGAGCCAAAAGGGTCATCATCT-3′	235
	R: 5′-GAGGGGCCATCCACAGTCTTCT-3′	

### Transfection of siRNA

JEG-3 cells were seeded in 6-well plates at a density of 1×10^5^/well, and transfected with CXCL12 siRNA, or CXCR4 siRNA, or non-targeting siRNA control in a final concentration of 125 nM using Lipofectamine 2000 reagent according to the manufacturer's recommendations. Sequences of siRNA used were indicated in Table 2. At 48 h post-transfection, JEG-3 cells were subjected to western blotting analysis.

**Table 2:** Sequences of small interfering RNA (siRNA).

Gene	Sequences
CXCL12	F: 5′-AUGGCUUUCGAAGAAUCGGCAUGGG-3′
	R: 5′-CCCAUGCCGAUUCUUCGAAAGCCAU-3′
CXCR4	F: 5′-AUACCAGGCAGGAUAAGGCCAACCA-3′
	R: 5′-UGGUUGGCCUUAUCCUGCCUGGUAU-3′

### Western blotting

JEG-3 cells were lysed in 1×SDS lysis buffer (50 mM Tris-HCl, pH 6.8, 2% SDS, 10% glycerol, 1 mM PMSF, and 1 mM Na_3_VO_4_), and performed as described previously [8]. Equal amount of total protein was loaded on an SDS-PAGE gel and transferred to PVDF membrane (Millipore, USA). After blocked with 5% BSA in PBS (containing 0.05% Tween 20), the membrane was incubated with specific primary antibodies, and followed by incubation with HRP-conjugated secondary antibodies. The protein bands of interest were visualized by fluorography by using an enhanced chemiluminescence system (Perfect Biotech, Shanghai, China).

### CXCL12 quantification by ELISA

Human trophoblast cells were treated with 1 μM CsA in 1640 complete medium supplemented with 10% FBS for 48 h, and the supernatants of cell cultures were harvested and tested by ELISA for CXCL12, according to the manufacturer's recommendation.

### Immunofluorescence

Primary human trophoblast cells were grown on eight-well chamber slide (R&D systems, Minneapolis, MN), fixed in 4% paraformaldehyde in PBS for 20 min, permeabilized with 0.1% Triton X-100 in PBS for 10 min, and blocked with 3% BSA in PBS for 30 min. Specimen was incubated with monoclonal EGFR antibody (1∶50 dilution in blocking solution) and monoclonal phosphorylated-EGFR antibody (1∶50 dilution in blocking solution) at 37°C for 3 h, followed by FITC conjugated secondary antibody (1∶50 dilution in blocking solution) and PE-conjugated secondary antibody (1∶50 dilution in blocking solution) 37°C for 1 h, and then counterstained with 4′,6-diamidino-2-phenylindole (DAPI) for 5 min at room temperature. Immunofluorescence was visualized by immunofluorescence microscope (Olympus BX51).

### BrdU cell proliferation assay

A colorimetric BrdU cell proliferation assay was used to evaluate cell proliferation according to the manufacturer's instructions. Human trophoblast cells were seeded in 96-well plates at a density of 2×10^4^/well. To evaluate cell proliferation, at 16 h before fixing cells, the thymidine analogue BrdU, was added to each well. Based on the incorporation of BrdU during DNA-synthesis, the amount of newly synthesized DNA and thus cell proliferation was detected by using a microplate absorbance reader (Bio-Rad iMark, Richmond, CA, USA) after applying anti-BrdU conjugated with peroxidase and enhancing a specific substrate reaction.

### Statistical analysis

Data are expressed as mean ± SEM, and statistical evaluation was performed by using one-way ANOVA followed a Dunnett test. Differences were accepted as significant at P<0.05.

## Results

### The effect of CsA on CXCL12 secretion in human trophoblast cells

**Figure 1:**
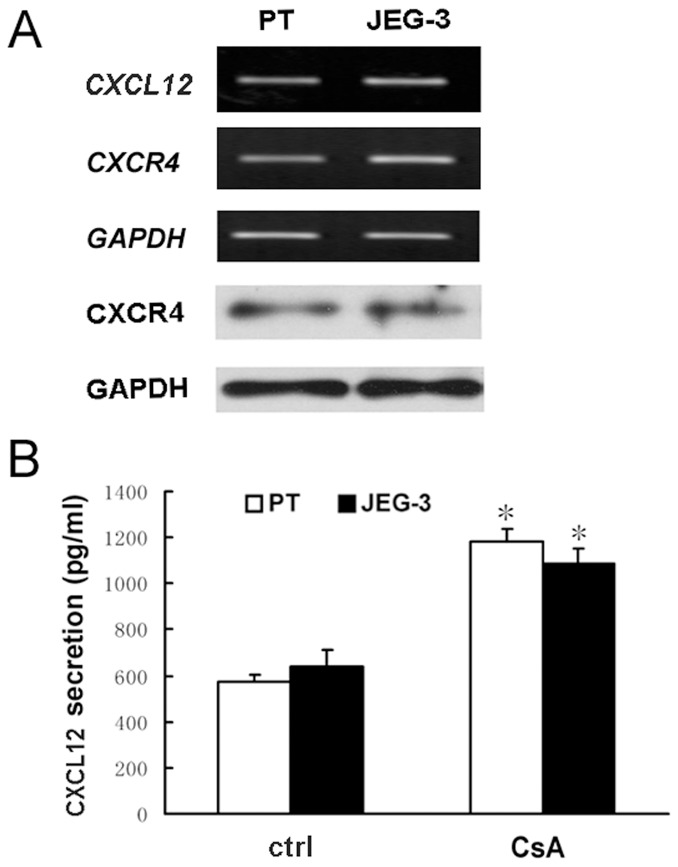
CsA promotes CXCL12 secretion in primary human trophoblast cells and JEG-3 cells. A : The mRNA expression of CXCL12 and CXCR4 in primary human trophoblast cells (labeled as PT) and JEG-3 cells was detected by RT-PCR (the upper panels). CXCR4 protein expression was detected by using western blotting (the lower panels). The GAPDH was used as a loading control. **B**: CXCL12 secretion in primary human trophoblast cells and JEG-3 cells was detected by ELISA. Human trophoblast cells were treated with 1 μM CsA in 1640 complete medium supplemented with 10% FBS for 48 h, and the culture medium was then collected and subjected to ELISA. Data are presented as mean ± SEM of three independent experiments. *P<0.05, compared to the control. Ctrl, control.

The mRNA expression of *CXCL12* and *CXCR4* in primary human trophoblast cells and JEG-3 cells was detected by RT-PCR, and the protein levels of CXCL12 and CXCR4 were further detected by ELISA assay and western blotting analysis, respectively. These data showed that both CXCL12 and its receptor CXCR4 were expressed in primary human trophoblast cells and JEG-3 cells (Fig. 1 A and B).

To detect the effect of CsA on CXCL12 secretion, primary human trophoblast and JEG-3 cells were treated with 1 µM CsA for 48 h, and then subjected to ELISA assay, respectively. As shown in Fig. 1 B, CsA induced a 2-fold increase in CXCL12 secretion in primary human trophoblast cells and JEG-3 cells.

### The effect of CXCL12 on EGFR phosphorylation in human trophoblast cells

**Figure 2:**
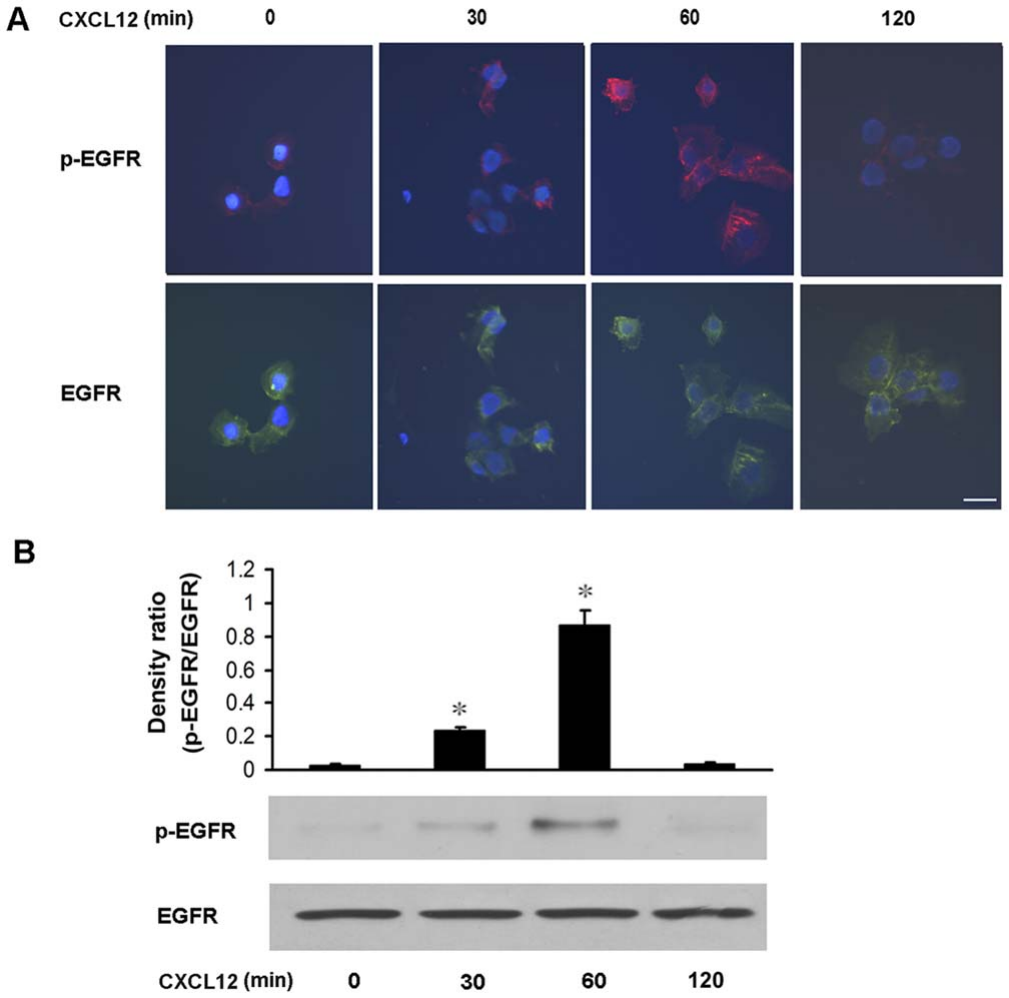
CXCL12 promotes the phosphorylation of EGFR in primary human trophoblast cells and JEG-3 cells. A : Recombinant human CXCL12 (20 ng/ml) was used to treat the primary human trophoblast cells for 30, 60, 120 min, respectively. The levels of phosphorylated EGFR and total EGFR were detected by immunofluorescence analysis. Scale bar, 25 μm. **B**: Recombinant human CXCL12 (20 ng/ml) was used to treat JEG-3 cells for 30, 60, 120 min, respectively. The phosphorylated and total levels of EGFR were analyzed by western blotting. A typical blot (the lower panels) and densitometry analysis of the ratio of phosphorylated EGFR to total EGFR (the upper panels) are shown. Data are presented as mean ± SEM of three independent experiments. *P<0.05, compared to the control.

We next detected the effect of CXCL12 on EGFR phosphorylation in primary human trophoblast cells. Primary human trophoblast cells were treated with recombinant human CXCL12 (20 ng/ml) for the indicated duration, and then the total and phosphorylated EGFR levels were detected by immunofluorescence analysis. As shown in Fig. 2 A, EGFR tyrosine phosphorylation was first detectable in 30 min after CXCL12 treatment, reached the maximal level at 60 min, and then decreased and returned to basal level within 120 min after the treatment.

We also tested whether recombinant human CXCL12 treatment resulted in EGFR activation in JEG-3 cells. JEG-3 cells were treated with CXCL12 (20 ng/ml) for the indicated duration, the total and phosphorylated levels of EGFR were detected by using western blotting analysis. Likewise, CXCL12 treatment caused EGFR phosphorylation starting in 30 min after treatment, increasing markedly in 60 min and returning to baseline in 120 min after the treatment (Fig. 2 B). These data imply that CXCL12 may trigger EGFR activation in human trophoblast cells.

### The role of CXCL12/CXCR4 axis in the CsA-induced EGFR phosphorylation in human trophoblast cells

**Figure 3:**
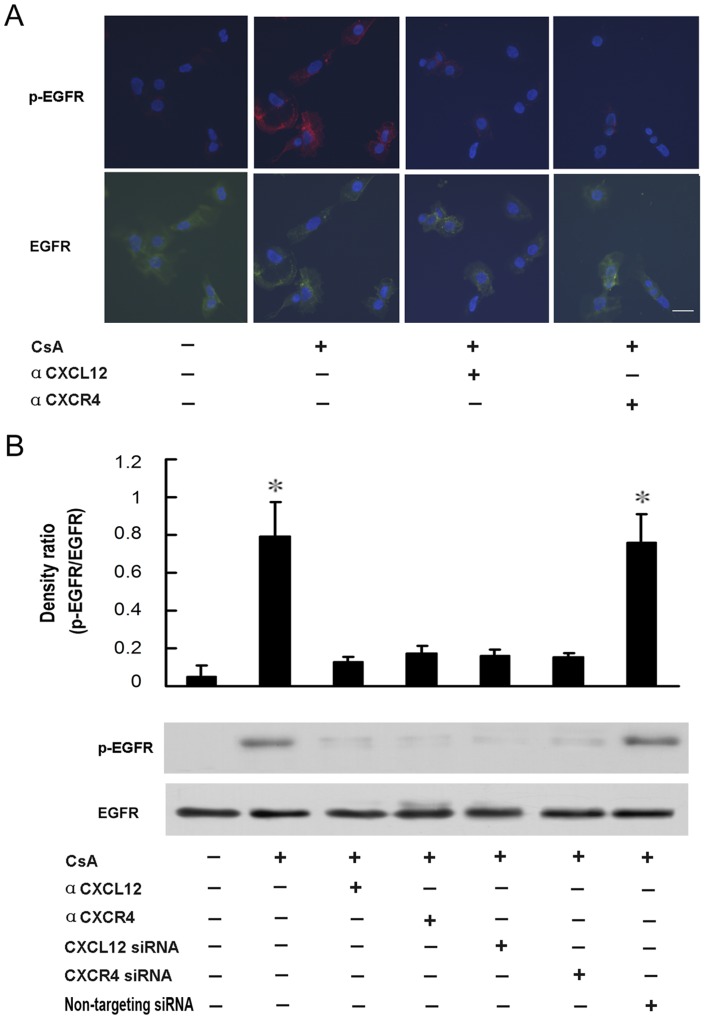
CsA promotes the phosphorylation of EGFR in a CXCL12/CXCR4 axis-dependent manner. **A**: Primary human trophoblast cells were treated with neutralizing antibody against CXCL12 (40 µg/ml) or CXCR4 (20 µg/ml) for 48 h, and then treated with 1 μM CsA in 1640 complete medium supplemented with 10% FBS for 1 h and subjected to immunofluorescence analysis to analyze the levels of phosphorylated EGFR and total EGFR. Scale bar, 25 μm. **B**: JEG-3 cells were transfected with CXCR4 siRNA (125 nM), or CXCL12 siRNA (125 nM), or non-targeting siRNA control, or treated with neutralizing antibody against CXCL12 (40 µg/ml) or CXCR4 (20 µg/ml) for 48 h, and then treated with 1 μM CsA in 1640 complete medium supplemented with 10% FBS for 1 h, and subjected to western blotting to analyze the levels of phosphorylated EGFR and total EGFR. A typical blot (the lower panels) and densitometry analysis of the ratio of phosphorylated EGFR to total EGFR (the upper panels) are shown. Data are presented as mean ± SEM of three independent experiments. *P<0.05, compared to the control.

As mentioned above, CsA promoted the phosphorylation of EGFR signaling pathway. To investigate the molecular mechanisms responsible for the CsA-induced EGFR phosphorylation, primary human trophoblast cells were pre-treated with the neutralizing antibody against CXCL12 or CXCR4 for 48 h, and then treated with CsA for 1 h, the total and phosphorylated levels of EGFR were analyzed by using immunofluorescence analysis. As shown in Fig. 3 A, CsA induced an increase in EGFR phosphorylation in primary human trophoblast cells, which could be completely abrogated by the neutralizing antibody against CXCL12 or CXCR4.

To further elucidate the role of CXCL12/CXCR4 axis in the CsA-induced EGFR phosphorylation, JEG-3 cells was pre-treated with the neutralizing antibody or siRNA against CXCL12 or CXCR4 for 48 h, then treated with CsA for 1 h, and the total and phosphorylated levels of EGFR were detected by using western blotting. As shown in Fig. 3 B, the CsA-induced EGFR phosphorylation was completely abolished in the presence of the neutralizing antibody or siRNA against CXCL12 or CXCR4, which provides further evidence that CXCL12/CXCR4 axis may be indispensable for the CsA-induced EGFR activation.

### The role of EGFR and its downstream ERK signaling pathway in the CsA-induced proliferation of human trophoblast cells

**Figure 4:**
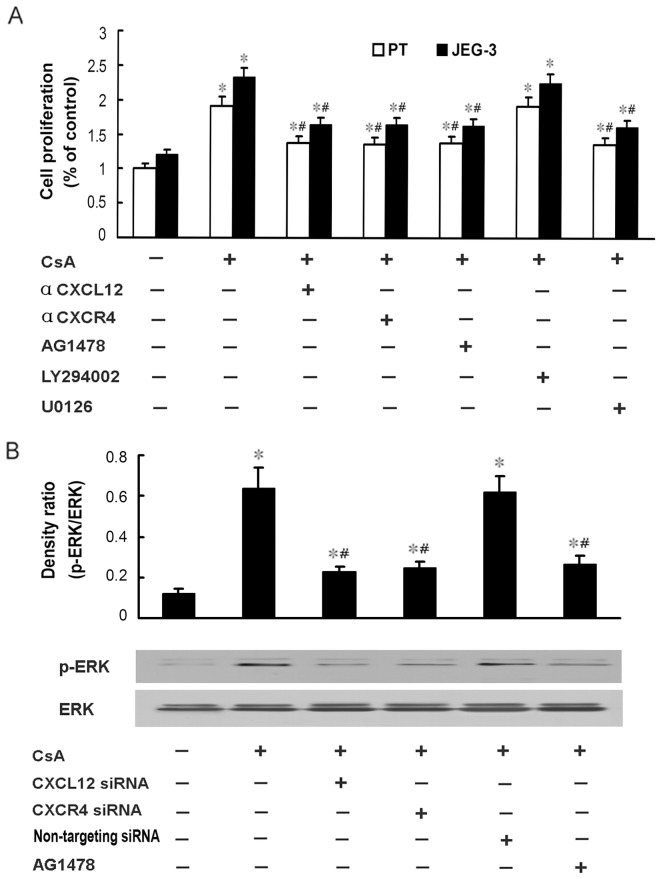
EGFR/ERK signaling pathway is involved in the CsA-induced proliferation of human trophoblast cells. **A**: Primary human trophoblast cells and JEG-3 cells were treated with 1 μM CsA and neutralizing antibody against CXCR4 (20 µg/ml) or CXCL12 (40 µg/ml), or U0126 (20 μM), or LY294002 (20 μM), or AG1478 (200 nM) for 48 h, and then subjected to BrdU cell proliferation assay. Data are presented as mean ± SEM of three independent experiments. *P<0.05, compared to the control. ^#^P<0.05, compared to CsA treatment group. **B**: JEG-3 cells were pretreated with siRNA against CXCL12 or CXCR4, or non-targeting siRNA control, or AG1478 for 48 h, and treated with 1 μM CsA in 1640 complete medium supplemented with 10% FBS for 1 h, and then subjected to western blotting to analyze the levels of phosphorylated ERK and total ERK. A typical blot (the lower panels) and densitometry analysis of the ratio of phosphorylated ERK to total ERK (the upper panels) are shown. Data are presented as mean ± SEM of three independent experiments. *P<0.05, compared to the control. ^#^P<0.05, compared to CsA treatment group.

It has been reported that Raf/MEK/ERK and PI3K/AKT signaling pathways represent the downstream targets of activated EGFR. We therefore investigate the involvement of the above signaling pathways in the CsA-induced proliferation of human trophoblast cells. As shown in Fig. 4 A, although JEG-3 cells demonstrated higher proliferation capacity than primary human trophoblast cells, CsA promoted the proliferation of these cells, compared to control cells. The CsA-induced proliferation could be markedly abrogated by the neutralizing antibody against CXCL12 or CXCR4, or the EGFR inhibitor AG1478, or the ERK inhibitor U0126. In contrast, the PI3K/PKB inhibitor LY294002 exerted no effect on the CsA-induced proliferation of human trophoblast cells. These findings indicate that CsA may promote the proliferation of human trophoblast cells through EGFR and ERK signaling pathway, rather than PI3K/AKT signaling pathway.

We further investigated whether the CsA-induced ERK activation was dependent on EGFR signaling pathway. JEG-3 cells were transfected with siRNA against CXCL12 or CXCR4, or treated with 200 nM AG1478 for 48 h, and then treated with CsA for 1 h. The total and phosphorylation levels of ERK were detected by western blotting analysis. Consistent with our previous report, CsA promoted the activation of ERK signaling pathway, which could be markedly abrogated by siRNA against CXCL12 or CXCR4, as well as AG1478 (Fig. 4 B). Together, these data suggest that CsA may promote the proliferation of human trophoblast cells through EGFR and its downstream ERK signaling pathway.

## Discussion

CsA is a widely used immunosuppressant agent to prevent from organ rejection and to treat certain autoimmune diseases. It has been reported that CsA treatment of adenocarcinoma cells results in striking morphological alterations, including membrane ruffling and numerous pseudopodial protrusions, increased cell motility, and anchorage-independent growth. However, this effect was independent of the immunosuppression of CsA [22]. Similarly, Han W *et* *al* have shown that CsA increases primary skin tumor growth in immunodeficient mice and promotes keratinocytes growth *in vitro* [23]. These findings imply that CsA modulates the biological behavior in various cells which does not concern its immunosuppressive action.

Previous data in our lab have demonstrated that CsA promotes the proliferation and invasion of human first-trimester trophoblast cells, and improves the outcome of pregnancy [5]–[7]. Further study in our group has found that CsA may promote the invasion through decreasing E-cadherin expression and increasing the expression of titin, MMP-2, and MMP-9 [6]–[8]. EGFR signaling pathway has been found to be involved in the CsA-induced invasion improvement in human trophoblast cells [8]. In placenta, EGFR is frequently highly activated, and controls the proliferation and invasion of human trophoblast cells [9].

**Figure 5:**
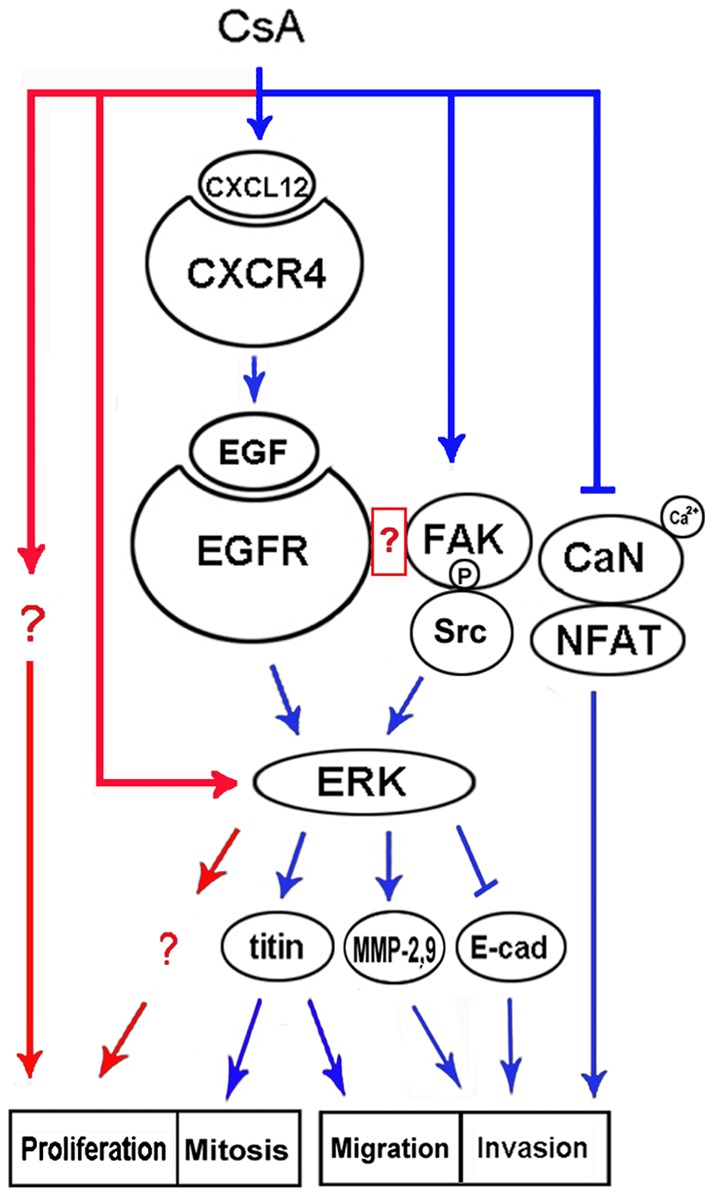
Schematic representation of intracellular signaling pathways in the modulation of CsA on the invasion and proliferation of human trophoblast cells. CsA promotes the invasion of human trophoblast cells via two independent signaling pathways, calcium/CaN/NFAT and EGFR/ERK. E-cadherin (labeled as E-cad), MMP-2, MMP-9, and titin may function as the downstream targets of EGFR/ERK signaling pathway in the CsA-induced invasion of human trophoblast cells. CsA may promote the activation of EGFR via CXCL12/CXCR4 axis. EGFR and its downstream ERK signaling pathway may also be involved in the CsA-induced proliferation of human trophoblast cells. Blue lines represent the signaling pathways proved to be involved in the regulation of CsA on the invasion of human trophoblast cells, and red lines represent the putative signaling pathways involved in the regulation of CsA on the proliferation of trophoblast cells.

In the present study we have demonstrated that CsA promotes EGFR phosphorylation in a CXCL12/CXCR4 axis-dependent manner. As mentioned before, CXCL12/CXCR4 axis is highly expressed in most normal tissues, and found to play diverse biological roles in different tissues. It is intriguing how CXCL12/CXCR4 axis promotes EGFR activation. EGFR has been shown to be activated by its direct natural ligands, EGF and TGF-á. In addition, EGFR is also activated by the secondary recruitment of other receptor types, such as G protein-coupled receptors (GPCRs). Stimulation of CXCR1/2 chemokine receptors by interleukin-8 has been shown to induce transient phosphorylation of EGFR, leading to the rapid activation of the p44/42 MAPK (also known as ERK1/2) in ovarian cancer cells [24]. Porcile found that CXCL12 induced EGFR phosphorylation in ovarian cancer cells [25]. Therefore, we hypothesize that CXCR4, as the member of the GPCRs family, may play a key role in the transactivation of the CsA-induced EGFR activation. Moreover, the CXCR4-triggered EGFR activation appear to be dependent on the stimulus of CXCL12 as either siRNA or neutralizing antibody against CXCL12 could effectively block the CsA-induced EGFR activation in primary human trophoblast cells and JEG-3 cells.

In the present study, we have shown that CsA may promote the proliferation of human trophoblast cells through EGFR/ERK signaling pathway. It is well established that ERK and PI3K/PKB signaling pathways are two key downstream molecules of EGFR. However, our present data show that PI3K/AKT signaling pathway is not responsible for the CsA-induced proliferation of human trophoblast cells. In contrast, ERK signaling pathway plays a pivotal role in the CsA-induced proliferation. However, in the epidermal keratinocytes, CsA inhibited PTEN transcription in an EGFR-dependent manner that ultimately led to the activation of AKT [23]. These data suggest that CsA downstream targets and network seem to be tissue specific.

In addition to EGFR/ERK signaling pathway, our previous study has shown the calcium/CaN/NFAT cascade is also responsible for the CsA-induced invasion in human trophoblast cells. However, the calcium/CaN/NFAT signaling pathway is not involved in the CsA-induced proliferation of human trophoblast cells [5]. In the present study, the neutralizing antibody against CXCR4 or CXCL12, or AG1478, or U0126 could not completely abrogated the CsA-induced proliferation, implying other signaling pathway may also be involved in the CsA-induced proliferation of human trophoblast cells (Figure 5).

Together, our previous and present data have shown that ERK cascade may play a key role in the regulation of CsA on the invasion and proliferation of human trophoblast cells. E-cadherin, MMP-2, MMP-9, and titin may function as the downstream targets of ERK in the CsA-induced invasion of human trophoblast cells. In the present study, CsA-induced ERK activation can not be entirely abrogated by siRNA against CXCR4 or CXCL12 as well as AG1478 (Figure 4). These data promote us to explore how other signaling pathway is involved in the activation of ERK and its downstream targets in the CsA-triggered proliferation of human trophoblast cells (Figure 5).

In fact, the intracellular network of CsA modulation may be far more complicated than it represents. Our unpublished data show that CsA promotes the activation of FAK/Src cascade. EGFR has been shown to be associated with FAK, and the transactivation of EGFR is necessary for FAK activation [26]. FAK activation triggers its autophosphorylation at tyrosine 397, allowing Src tyrosine kinase to bind to FAK and generating a FAK/Src signaling complex. However, the association of FAK with EGFR is indirect, and it is recently identified that alternate-spliced isoform of steroid receptor coactivator-3 (SRC-3) may act as an EGFR-FAK bridging protein [27]. Further study is required to elucidate whether CsA initiates FAK/Src activation in an EGFR-dependent manner (Figure 5).

The proliferation and invasion of human trophoblast cells is crucial for the establishment of a successful pregnancy. Aberrant invasion and proliferation of human trophoblast cells is thought to result in the pathogenesis of pregnancy-associate disorders including pre-eclampsia, miscarriage, and trophoblastic disease [28]–[30]. It still need more research to investigate the safety and long-term consequences of CsA treatment before it is developed to a potential drug for pregnancy complications although we have a large body of evidence shows that CsA induces the immune tolerance at the maternal-fetal interface, and improves biological behavior of trophoblast cells required for a successful pregnancy.
